# Non-invasive Characterization of Human AV-Nodal Conduction Delay and Refractory Period During Atrial Fibrillation

**DOI:** 10.3389/fphys.2021.728955

**Published:** 2021-10-28

**Authors:** Mattias Karlsson, Frida Sandberg, Sara R. Ulimoen, Mikael Wallman

**Affiliations:** ^1^Department of Systems and Data Analysis, Fraunhofer-Chalmers Centre, Gothenburg, Sweden; ^2^Department of Biomedical Engineering, Lund University, Lund, Sweden; ^3^Department of Medical Research, Vestre Viken Hospital Trust, Bærum Hospital, Drammen, Norway

**Keywords:** atrial fibrillation, atrioventricular node, rate control, mathematical modeling, genetic algorithm, ECG, cardiac electrophysiology

## Abstract

During atrial fibrillation (AF), the heart relies heavily on the atrio-ventricular (AV) node to regulate the heart rate. Thus, characterization of AV-nodal properties may provide valuable information for patient monitoring and prediction of rate control drug effects. In this work we present a network model consisting of the AV node, the bundle of His, and the Purkinje fibers, together with an associated workflow, for robust estimation of the model parameters from ECG. The model consists of two pathways, referred to as the slow and the fast pathway, interconnected at one end. Both pathways are composed of interacting nodes, with separate refractory periods and conduction delays determined by the stimulation history of each node. Together with this model, a fitness function based on the Poincaré plot accounting for dynamics in RR interval series and a problem specific genetic algorithm, are also presented. The robustness of the parameter estimates is evaluated using simulated data, based on clinical measurements from five AF patients. Results show that the proposed model and workflow could estimate the slow pathway parameters for the refractory period, $R_{min}^{SP}$ and Δ*R*^*SP*^, with an error (mean ± std) of 10.3 ± 22 and −12.6 ± 26 ms, respectively, and the parameters for the conduction delay, $D_{min,tot}^{SP}$ and $\Delta D_{tot}^{SP}$, with an error of 7 ± 35 and 4 ± 36 ms. Corresponding results for the fast pathway were 31.7 ± 65, −0.3 ± 77, 17 ± 29, and 43 ± 109 ms. These results suggest that both conduction delay and refractory period can be robustly estimated from non-invasive data with the proposed methodology. Furthermore, as an application example, the methodology was used to analyze ECG data from one patient at baseline and during treatment with Diltiazem, illustrating its potential to assess the effect of rate control drugs.

## 1. Introduction

Atrial fibrillation (AF) is the most widespread sustained cardiac arrhythmia with an estimated prevalence of 2–4% in the adult population (Benjamin et al., 2019). During AF, the electrical activity in the atria is highly disorganized, leading to a rapid and irregular ventricular rhythm. In order to reduce these effects, rate control drugs constitute one of the primary therapeutic options (Hindricks et al., 2020). These drugs are not designed to terminate AF, but rather to lower the heart rate. They do this by modulating the conduction through the AV node, preventing some electrical signals emanating from the atria from being transmitted to the ventricles, thereby reducing the ventricular activation rate. Thus, rate control is often sufficient to improve AF-related symptoms (Hindricks et al., 2020). The choice of first-line rate control drugs can vary between beta-blockers and non-dihydropyridine calcium channel blockers, with digoxin as a second-line option (Hindricks et al., 2020). However, the current method of finding the best treatment for a given patient is largely based on trial and error (Hindricks et al., 2020). Thus, patient specific characterization of AV node properties would be beneficial to achieve optimal rate control.

Functionally, the AV node consists of two pathways, connected to each other before entering the bundle of His (Kurian et al., 2010). The two pathways are referred to as the slow pathway (SP) and the fast pathway (FP), where the FP conducts impulses faster than SP but has a longer refractory period. During sinus rhythm, the impulses are typically conducted through the FP due to its faster conduction rate. During AF, however, conduction may alternate between SP and FP as a result of the rapid arrival of atrial impulses. This, together with concealed conduction, i.e., impulses inside the AV node that do not lead to ventricular activation but still affect the conduction characteristics of following impulses, gives rise to the complex blocking and delay behavior the AV node has been shown to possess.

In order to understand this blocking and delay behavior, mathematical modeling has become an increasingly important tool. Several models of the AV node and its function during AF have previously been proposed, including various descriptions of the conduction delay (Jørgensen et al., 2002; Mangin et al., 2005; Climent et al., 2011) and the refractory period (Rashidi and Khodarahmi, 2005). A model for simulating the ventricular activation capable of replicating both conduction delay and refractory period during AF was proposed by Lian et al. (2006). Another model capable of replicating both conduction delay and refractory period, based on the action potential of the AV node cells and modeled by ordinary differential equations, was proposed by Inada et al. (2009).

However, none of these models were developed with the purpose of ECG based estimation of AV node parameters on a patient specific basis. The models presented in Rashidi and Khodarahmi (2005) and Lian et al. (2006) did not fit parameter values to data, the models presented in Climent et al. (2011) and Inada et al. (2009) were fitted to data from rabbits. The models presented in Jørgensen et al. (2002) and Mangin et al. (2005) were fitted to AF patients, but invasive data was required. To make a model useful in a clinical setting, it should ideally allow for fitting to non-invasive data such as surface electrocardiogram (ECG). A statistical model developed for estimation of AV node parameters from ECG data during AF was first presented in Corino et al. (2011). This model has later been updated and proven to replicate patient specific histograms of the time series between two successive R waves on the ECG (RR interval series) extracted from ECG data, as well as to assess the effect of rate control drugs on the AV node (Henriksson et al., 2015). It is a lumped model structure that still accounts for concealed conduction, relative refractoriness, and dual pathways. However, it lumps conduction delay and refractory period together, making the estimated model parameters difficult to interpret.

In this work we present a network model of the AV node, able to estimate patient specific conduction delay and refractory period from ECG, building on previous work presented in Wallman and Sandberg (2018). The model consists of interconnected nodes forming two pathways, providing a balance between complexity and computational efficiency, and represents both spatial and temporal dynamics of the AV-node. With novel additions to the model structure by including effects from the bundle of His and Purkinje fibers, as well as a tailored workflow taking advantage of dynamics in the data, the model allows for estimation of parameters governing both refractory period and conduction delay in a robust manner from non-invasive data during AF. The ultimate aim of this work is to monitor and predict the outcome of treatment with rate control drugs in clinical settings to assist in treatment selection. In order to do this, a robust characterization of the AV node is needed, and thus the purpose of this study is to: (1) Describe and motivate the model; (2) Present a tailored workflow for estimation of parameters; (3) Demonstrate that presented combination of model and workflow leads to robust parameter estimates that mimic measured data well.

## 2. Materials and Methods

The model of the AV node will be explained in section 2.1, followed by a description of the data used to evaluate said model in sections 2.2 and 2.3. In section 2.4, the methodology for model parameter estimation is explained; which combined with the optimization algorithm described in section 2.5 constitutes the workflow.

### 2.1. Network Model of the Human AV Node

**Figure 1:**
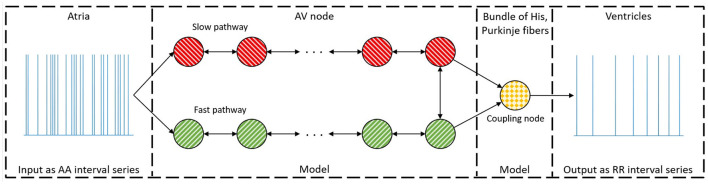
A schematic representation of the proposed model. The arrow indicates the direction an impulse can conduct, and the colors represent nodes with the same parameter sets. For simplicity, only a subset of the ten nodes in each pathway are showed.

The model of the AV node, shown in Figure 1, consists of a network of nodes and is based on the model presented in Wallman and Sandberg (2018). The model consists of two pathways, representing the SP and the FP, connected with a coupling node. Each pathway is modeled with 10 nodes, where each node corresponds to a localized part of the AV node. Each node can block incoming impulses or send them through adding a conduction delay. All nodes but the coupling node sends impulses to all other nodes connected to it, whereas the coupling node only receives impulses. A new refractory period [*R*_*i*_(*n*)] and conduction delay [*D*_*i*_(*n*)] are calculated every time a node (*i*) receives a new impulse (*n*). The refractory period and conduction delay are based on the stimulation history of the node and are described using exponential functions. These exponential functions have previously been used to fit AV node characteristics (Shrier et al., 1987; Lian et al., 2006; Wallman and Sandberg, 2018), and can be seen in Equations (1–3).


(1) $$\begin{array}{l} R_{i}\left(n\right)=R_{min}+\Delta R\left(1-e^{-{\tilde{t}}_{i}\left(n\right)/\tau_{R}}\right) \end{array}$$

(2) $$\begin{array}{l} D_{i}\left(n\right)=D_{min}+\Delta De^{-{\tilde{t}}_{i}\left(n\right)/\tau_{D}} \end{array}$$

(3) $$\begin{array}{l} {\tilde{t}}_{i}\left(n\right)=t_{i}\left(n\right)-t_{i}\left(n-1\right)-R_{i}\left(n-1\right) \end{array}$$

Here ${\tilde{t}}_{i}\left(n\right)$ refers to diastolic interval preceding impulse *n*, *t*_*i*_(*n*) the arrival time of impulse *n* at node *i*, and *t*_*i*_(*n* − 1) and *R*_*i*_(*n* − 1) the arrival time and refractory period of impulse *n* − 1 at node *i*, respectively. If ${\tilde{t}}_{i}\left(n\right)$ is negative, the node will still be in its refractory period and thus the impulse will be blocked. The model parameters defining minimum refractory period, *R*_*min*_; maximum prolongation of refractory period, Δ*R*; time constant τ_*R*_; minimum conduction delay, *D*_*min*_; maximum prolongation of conduction delay, Δ*D*; and the time constant τ_*D*_, are assumed to be fixed for the nodes in the SP and FP, respectively.

The coupling node models the total refractoriness and conduction delay introduced by the connection between the AV node and the bundle of His, the Purkinje fibers, and the bundle of His. This node has a separate set of parameters, representing separate functional properties, and will be denoted the His and Purkinje (HP) node. The refractory period for the Purkinje fibers is assumed to not affect the ventricular activation during AF. Thus, the whole refractory period for the HP node is determined by the bundle of His. However, the conduction delay for the HP node is viewed as the time it takes an impulse to travel from the start of the bundle of His to the end of the Purkinje fibers. The conduction delay from the start of the bundle of His until the end of the Purkinje fibers has clinically been showed to have a mean of 60 ms with a standard deviation of 10 ms for patients suffering from AF (Deshmukh et al., 2000). Thus, the conduction delay for the HP node is fixed at 60 ms. The HP node's refractory period is estimated by the mean of the ten shortest RR intervals, *RR*_*min*_.

This results in 12 free parameters for the proposed model, denoted as a parameter vector $\theta =[R_{min}^{FP},\;\Delta R^{FP},$ $\tau_{R}^{FP},\;R_{min}^{SP},\;\Delta R^{SP},\;\tau_{R}^{SP},\;D_{min}^{FP},\;\Delta D^{FP},\;\tau_{D}^{FP},\;D_{min}^{SP},\;\Delta D^{SP},\;\tau_{D}^{SP}]$. It is assumed that the first node of each pathway is simultaneously stimulated for incoming impulses from the atria. The model can then be used to produce a RR interval series with minimal computational demands using a modified version of Dijkstra's algorithm (Wallman and Sandberg, 2018). A link to the code for the model together with a basic user example can be found at section 5. The total minimum conduction delay and maximum prolongation, defined as $D_{min,tot}^{FP}=N_{n}D_{min}^{FP}$; $\Delta D_{tot}^{FP}=N_{n}\Delta D^{FP}$; $D_{min,tot}^{SP}=N_{n}D_{min}^{SP}$; $\Delta D_{tot}^{SP}=N_{n}\Delta D^{SP}$; where *N*_*n*_ = 10 are the number of nodes in each pathway, are introduced for convenience of presentation.

### 2.2. ECG Data

This study was based on ambulatory ECG data from the RATe control in Atrial Fibrillation (RATAF) study, which is approved by the regional ethics committee and the Norwegian medicines agency and was conducted in accordance with the Helsinki Declaration (Ulimoen et al., 2013). The RATAF study contains 24-h Holter recordings of 60 patients under baseline and during treatment with four different rate reducing drugs. All patients had permanent AF, no heart failure or symptomatic ischemic heart disease, an age of 71±9 (mean ± std), and 70% were men. To evaluate the presented model, we selected 15 min ECG segments, one for each of five patients, obtained under baseline conditions between 1:00 and 3:00 pm. These five patients were selected to be representative for the whole data set, with varying RR interval series characteristics and an average heart rate ranging between 63 and 140 bpm. In addition, corresponding ECG data obtained during treatment with Diltiazem was also used for one of the five patients.

The RR interval series were extracted from the ECG signals by first detecting the R peaks, before removing RR intervals preceding and following ectopic beats identified based on heartbeat morphology (Lagerholm et al., 2000). Along with this, the mean arrival rate of the atrium-to-atrium (AA) intervals was estimated from the f-waves in the ECG by first extracting the atrial activity from the ECG using spatiotemporal QRST cancellation (Stridh and Sornmo, 2001), before tracking the atrial fibrillatory rate (AFR) using a method based on a hidden Markov model (Sandberg et al., 2008). Finally, correction of the atrial fibrillatory rate by taking the atrial depolarization time into account was used to obtain an estimate of the arrival rate. Here, we denote the true mean arrival rate λ, and the estimated mean arrival rate $\hat{\lambda }$. One value of $\hat{\lambda }$ was obtained for each ECG segment (Corino et al., 2013).

### 2.3. Simulated Data

Simulated data were created by fitting the model to the RR interval series from the five patients, cf. section 2.5, and using the resulting estimated model parameters to simulate an RR interval series of 20 min. The sequence of atrial impulses arriving to the AV node, and thus the input to the model, were simulated using a Poisson process with the mean arrival rate set to the value of $\hat{\lambda }$ estimated for each patient (Corino et al., 2011; Henriksson et al., 2015). The parameter values used for the simulated data, along with average heart rate of the simulated RR interval series, are summarized in Table 1.

**Table 1:** Characteristics of the data extracted from ECG and the simulated data, respectively, for all five patients.

**Parameter**	**Patient 1**	**Patient 2**	**Patient 3**	**Patient 4**	**Patient 5**
**MEASURED DATA**					
Average HR (ms)	76.4	62.7	90.6	111.9	139.9
$\hat{\lambda }$ (Hz)	8.45	9.13	6.73	9.03	10.04
**SIMULATED DATA**					
Average HR (bpm)	75.3	62.3	93.1	110.5	139.5
λ (Hz)	8.45	9.13	6.73	9.03	10.04
SP ratio (%)	54	60	85	77	92
$R_{min}^{FP}$ (ms)	210	390	379	465	378
Δ*R*^*FP*^ (ms)	516	475	594	1.47	383
$\tau_{R}^{FP}$ (ms)	168	217	222	113	145
$R_{min}^{SP}$ (ms)	205	313	280	257	287
Δ*R*^*SP*^ (ms)	469	422	233	0.00	103
$\tau_{R}^{SP}$ (ms)	220	40	204	172	227
$D_{min}^{FP}$ (ms)	4.77	1.13	1.44	9.05	6.43
Δ*D*^*FP*^ (ms)	11.2	20.6	16.0	20.3	34.4
$\tau_{D}^{FP}$ (ms)	155	237	40.0	40.0	145
$D_{min}^{SP}$ (ms)	21.1	25.4	21.7	16.0	20.2
Δ*D*^*SP*^ (ms)	51.9	15.1	4.62	3.74	2.47
$\tau_{D}^{SP}$ (ms)	89.9	232	166	91.1	165

### 2.4. Model Parameter Estimation

To evaluate how well the model matches the extracted RR interval series, a fitness function comparing the model output to the RR interval series is used. In order to take the dynamics of the RR interval series into account, the Poincaré plot is used as a basis for the fitness function. The Poincaré plot is a scatter plot of successive pairs of RR intervals. To use the Poincaré plot as a fitness function, the RR interval series is binned into two dimensional bins centered between 250 and 1,800 ms in steps of 50 ms, resulting in *N* = 961 bins. The error function is computed according to Equation (4).


(4) $$\begin{array}{l} \epsilon =\frac{1}{N}\overset{N}{\underset{i=1}{\sum }}\left({\left(x_{i}-{\tilde{x}}_{i}\right)}^{2}/\sqrt{{\tilde{x}}_{i}}\right) \end{array}$$

**Figure 2:**
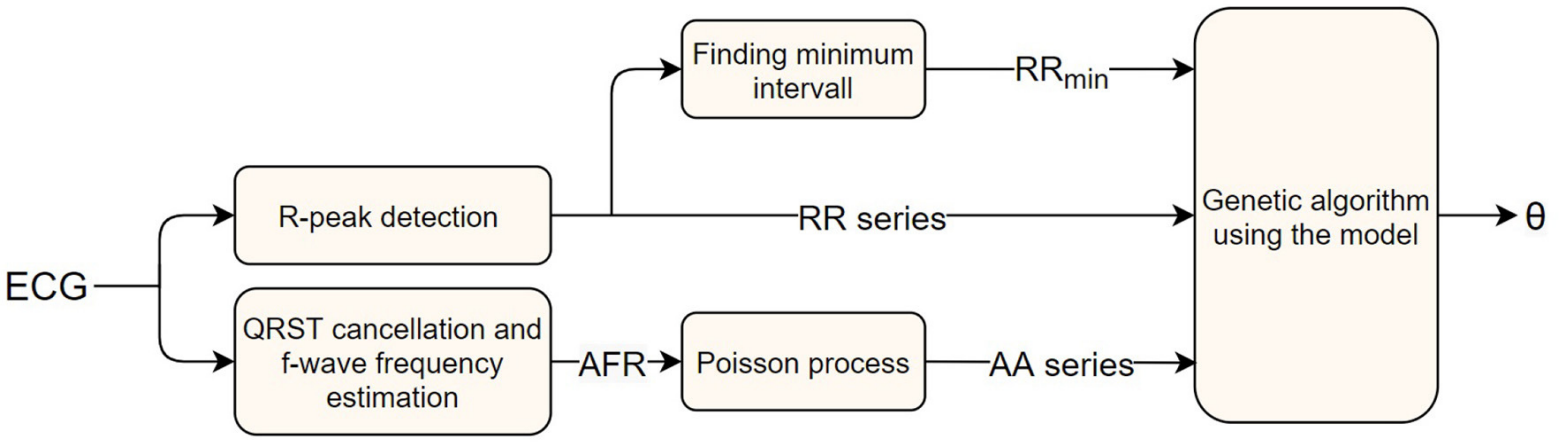
A block diagram of the AV node model parameter estimation workflow, starting with a measured ECG signal and ending with estimated parameters.

Here ϵ is the error value, and $\tilde{x_{i}}$ and *x*_*i*_ the number of RR intervals, in the *i*-th bin, of the measured data and model output, respectively. The normalization by $\sqrt{{\tilde{x}}_{i}}$ is introduced to avoid bins with a large number of data points to dominate the optimization. The square root is used as a trade-off between no normalization, making the bins with a large number of data points dominate, and normalization with the whole measured bin counts, making the accuracy of every bin have the same weight regardless of how much of the data are in that bin. A schematic representation of the parameter estimation process can be seen in Figure 2.

### 2.5. Genetic Algorithm

**Figure 3:**
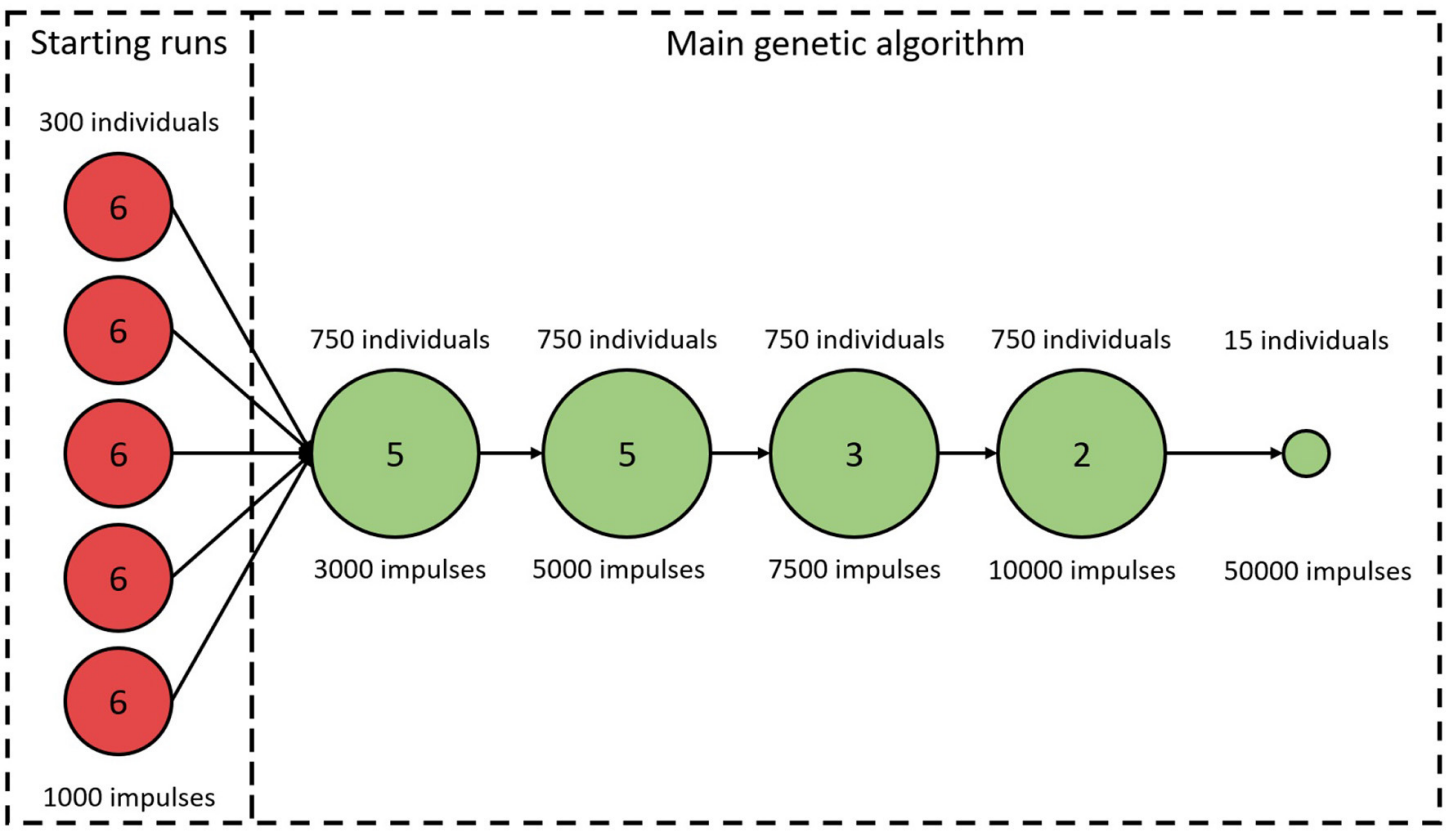
A schematic representation of the genetic algorithm. Circles represent stages of the algorithm with constant number of individuals and *L*_*RR*_. Numbers in circles correspond to the number of iterations before proceeding to the next stage. The last stage is always used, even if the GA terminates early.

An initial study of how ϵ varies with varying model parameter values revealed a highly chaotic structure with a large number of local minima. This prompted us to minimize ϵ using a genetic algorithm (GA). A brief description of the algorithm is given below, with more detailed information in the Supplementary Section 1. Due to the high dimensional parameter space and the risk of premature convergence early in the optimization, a variant of an island model was used (Wahde, 2008). A schematic representation of the GA is shown in Figure 3. As visible in the figure, the full GA can be divided into two sections. The first section consists of five separate GA. This was implemented by restarting the algorithm five times with 300 individuals in each generation. The individuals in each starting run were initialized using a latin hypercube sampling in the ranges: $\left\{R_{min}^{SP},R_{min}^{FP}\right\}\in \left[250,600\right]\;ms$; {Δ*R*^*SP*^, Δ*R*^*FP*^} ∈ [0, 600] *ms*, $\left\{\tau_{R}^{SP},\tau_{R}^{FP}\right\}\in \left[50,300\right]\;ms$; $\left\{D_{min}^{SP},D_{min}^{FP}\right\}\in \left[0,30\right]\;ms$; {Δ*D*^*SP*^, Δ*D*^*FP*^} ∈ [0, 75] *ms*; $\left\{\tau_{D}^{SP},\tau_{D}^{FP}\right\}\in \left[50,300\right]\;ms$. These starting runs last for six generations, and after each run the best 150 of the individuals are saved and used in the second section, the main GA. Thus, the main GA uses a population of 750 individuals in each generation. For both the starting runs and the main GA, the 2.5% fittest individuals in each generation survives into the next generation unchanged, whereas the remaining individuals are created via tournament selection, two-point crossover, and creep mutation (Wahde, 2008). In order to avoid premature convergence, both incest prevention in the form of mating restriction between too similar individuals during crossover, and a varying mutation rate depending on the diversity of the individuals in each generation were implemented (Wahde, 2008). This process of selection, crossover, and mutation is then continued until termination. The termination of the starting runs always occurs after six generations. The termination for the main GA occurs either when ϵ for the fittest individual in each generation does not change for three generations, or when 15 generations have been run. The fittest individual for the *k*-th generation, $\hat{\epsilon_{k}}$, is deemed to have changed if the difference between $\bar{\hat{\epsilon }}\left(k\right)$ and $\bar{\hat{\epsilon }}\left(k-2\right)$, seen in Equation (5), is lower than 25.


(5) $$\begin{array}{l} \bar{\hat{\epsilon }}\left(k\right)=\frac{{\hat{\epsilon }}_{k}+{\hat{\epsilon }}_{k-1}+{\hat{\epsilon }}_{k-2}}{3} \end{array}$$

**Figure 4:**
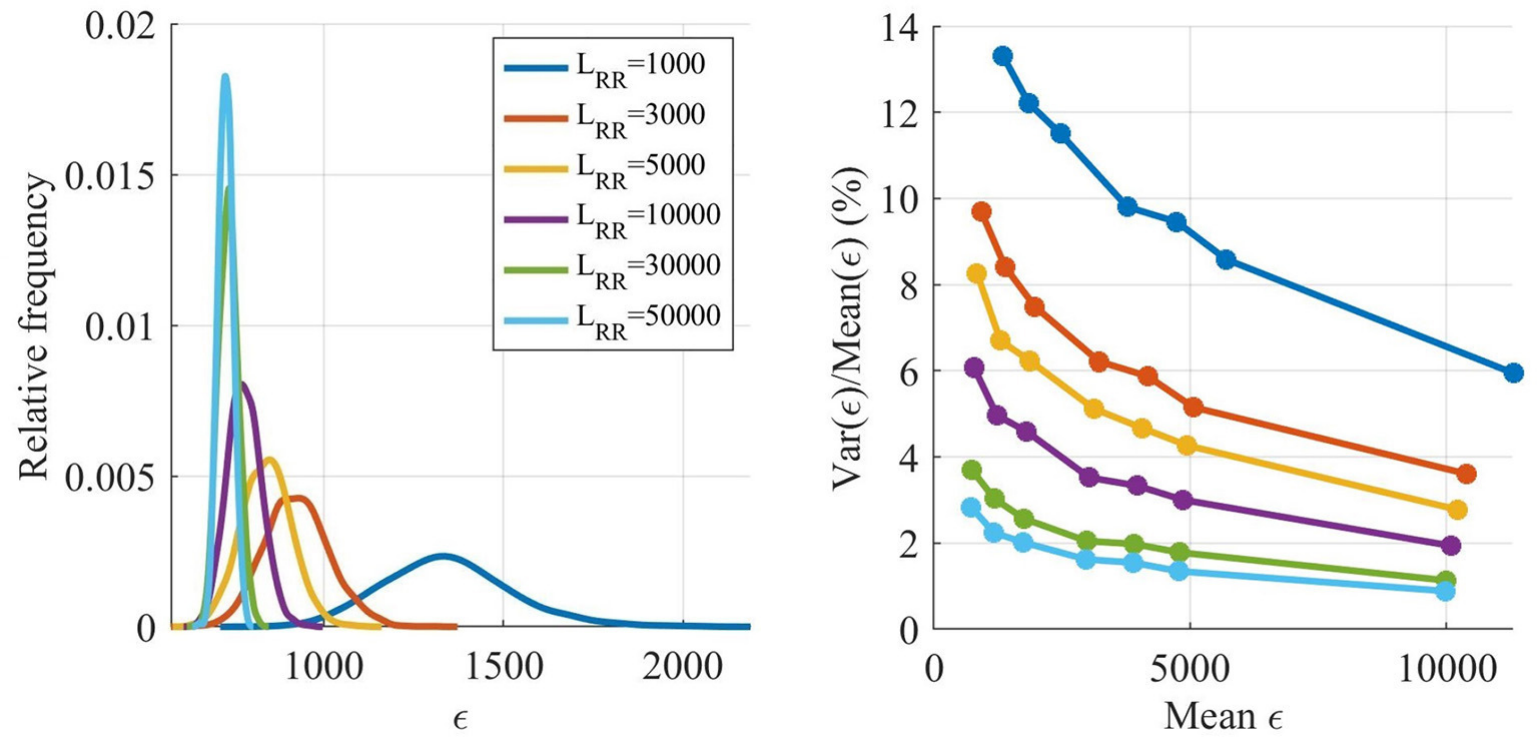
Estimated distribution of ϵ as a function of *L*_*RR*_ **(left)**. Variance of ϵ divided by mean of ϵ as a function of the mean of ϵ **(right)**.

As described in section 2.3, a Poisson process with mean arrival rate $\hat{\lambda }$ was used as input to the model, and due to the stochastic nature of the Poisson process, ϵ varies between realizations. The magnitude of this variation was analyzed by finding a parameter set replicating the extracted RR interval series from patient 3 well, before simulating that parameter set with different lengths of the resulting RR interval series, *L*_*RR*_, as seen in Figure 4, left panel. Each *L*_*RR*_ was simulated 1,000 times. Moreover, six more parameter sets with increasing ϵ were also simulated 1,000 times with the same *L*_*RR*_, as seen in Figure 4, right panel.

The ϵ variation is decreasing with larger *L*_*RR*_, however, the running time for the model is linearly increasing with *L*_*RR*_, and thus shorter outputs are preferable. The variation of ϵ is not as important early in the optimization since the variation relative ϵ is smaller for larger ϵ, see Figure 4, right panel. However, after several generations most of the ϵ for individuals found by the GA are low, and thus the variability in ϵ has a larger impact on the algorithm. Therefore, *L*_*RR*_ is increased throughout the optimization.

As seen in Figure 3, the *L*_*RR*_ for all generations in the starting runs were 1,000 impulses. For the main GA, the first five generations used a *L*_*RR*_ of 3,000 impulses, the following five generations a *L*_*RR*_ of 5,000 impulses, followed by three generations with length of 7,500 impulses, before ending with two 10,000 impulses long generations. To obtain a robust estimate of $\bar{\hat{\epsilon }}\left(k\right)$, the individual with the best fit in each generation is evaluated again with a *L*_*RR*_ of 10,000. After termination for the main GA, the 15 fittest individuals were tested again, with a *L*_*RR*_ of 50,000; this in order to select the fittest individual with a low variation in ϵ.

## 3. Results

The RR interval series extracted from the ECG along with the simulated data, cf. sections 2.2 and 2.3, are used to evaluate the proposed methodology. In section 3.1, the proposed approach for optimization is compared to using only the main GA with fixed *L*_*RR*_. The robustness and precision of the parameter estimation are evaluated using simulated data in section 3.2. Further, the robustness of the estimates is set in perspective by using the model to estimate AV node characteristics for one of the patients during both baseline and under influence of the calcium channel blocker drug Diltiazem. In section 3.3, the proposed model is compared to the model presented in Wallman and Sandberg (2018).

### 3.1. Genetic Algorithm

**Figure 5:**
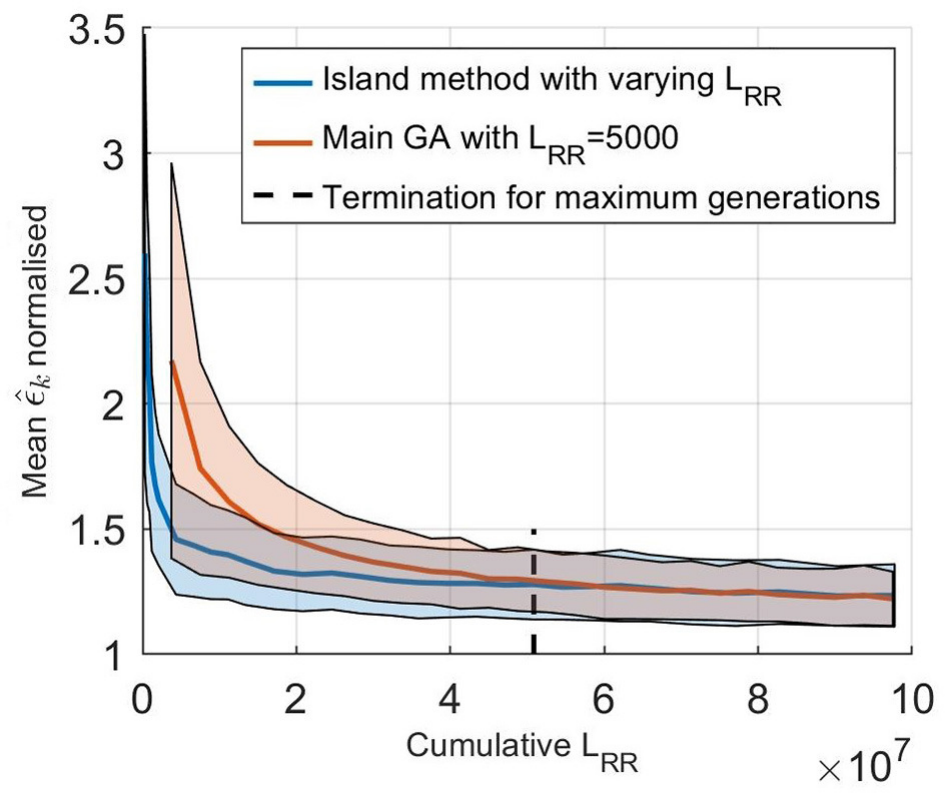
(solid line) Mean normalized ${\hat{\epsilon }}_{k}$ of 100 optimizations of the five set of patient data as a function of cumulative *L*_*RR*_ for (blue) the island start optimization with varying *L*_*RR*_ and (orange) only the main GA with a fix *L*_*RR*_ at 5,000. The shaded background represents one standard deviation. Here, ${\hat{\epsilon }}_{k}$ is normalized with the best ϵ found for each patient, to account for the fact that the model can not fit each RR interval series equally well.

The effect of using an island based start together with varying *L*_*RR*_ was evaluated by comparing it to using only the main GA, as described in section 2.5, with *L*_*RR*_ fixed at 5,000. The initialization for this fixed GA was the same as for the starting runs, a latin hypercube sampling in the same ranges, and the population size was again 750. Performances of the two methods were evaluated by comparing the error value of the fittest individual for each generation, ${\hat{\epsilon }}_{k}$ with the cumulative *L*_*RR*_ used for the evaluations, i.e., the accumulated total number of impulses in each generation. For the different starting runs, all runs were computed in parallel so that ${\hat{\epsilon }}_{k}$ during this stage is the lowest value out of all the five starting runs. The average results from comparing the two versions of the GA on all five patients, each 100 times, are shown in Figure 5. From this it is possible to see that a lower ${\hat{\epsilon }}_{k}$, and thus a better fit to the RR interval series, can be found in less computational time using the proposed methodology. For reference, estimating the parameters for one patient using a single core on a standard desktop computer (Intel® Core™ i7-6600U Processor, @ 2.60GHz) requires on average 20 min, with variations due to the different terminating requirements for the GA. It is also possible to see that the termination criteria for a maximum number of generations stated in section 2.5 is typically achieved after the GA has converged.

### 3.2. Parameter Estimation Robustness

Simulated RR interval series were used to evaluate the robustness of the model parameter estimates. The results from optimizing the model 200 times for the five simulated RR interval series can be seen in Table 2, where the mean and standard deviation for each of the 12 estimated parameters, for each of the five patients, are listed. Moreover, the mean error, defined as the difference between the mean value of the estimated parameter and the ground truth, averaged over the five patients, are also listed. Furthermore, the mean and standard deviation of the error normalized with respect to the parameter ranges, cf. section 2.5, are presented. From the SP ratio it is evident that the SP is used more for transmission, and from the normalized error, it is evident that the parameters associated with the SP are more robustly estimated. The histogram and Poincaré plots for the five simulated patients with the transmission pathway for each RR interval marked out can be seen in Supplementary Section 3, together with the simulated histograms showing the effect of changes to λ.

**Table 2:** The mean parameter values ± standard deviation of 200 optimizations for the five simulated data sets, together with the mean error ± mean standard deviation for each parameter.

**Parameter**	**Patient 1**	**Patient 2**	**Patient 3**	**Patient 4**	**Patient 5**	**Error**	**Normalized error (%)**
$R_{min}^{FP}$ (ms)	311 ± 104	394 ± 53	430 ± 49	424 ± 45	419 ± 72	31.7 ± 65	7.9 ± 16
Δ*R*^*FP*^ (ms)	436 ± 74	495 ± 57	479 ± 55	164 ± 131	393 ± 69	-0.3 ± 77	-0.1 ± 12
$\tau_{R}^{FP}$ (ms)	184 ± 38	211 ± 35	168 ± 39	183 ± 63	167 ± 53	9.4 ± 45	3.6 ± 17
$R_{min}^{SP}$ (ms)	225 ± 17	369 ± 71	271 ± 11	247 ± 8	281 ± 5	10.3 ± 22	2.6 ± 6
Δ*R*^*SP*^ (ms)	430 ± 26	358 ± 60	247 ± 14	28 ± 20	101 ± 4	-12.6 ± 26	-1.9 ± 4
$\tau_{R}^{SP}$ (ms)	201 ± 29	56 ± 10	216 ± 26	204 ± 55	198 ± 41	2.2 ± 32	0.8 ± 12
$D_{min,tot}^{FP}$ (ms)	65 ± 31	36 ± 22	53 ± 21	69 ± 39	92 ± 38	17 ± 29	5.7 ± 10
$\Delta D_{tot}^{FP}$ (ms)	188 ± 92	273 ± 9.6	193 ± 95	248 ± 119	336 ± 145	43 ± 109	5.7 ± 15
$\tau_{D}^{FP}$ (ms)	132 ± 48	150 ± 43	133 ± 47	135 ± 47	154 ± 47	17 ± 46	7.1 ± 19
$D_{min,tot}^{SP}$ (ms)	184 ± 36	245 ± 25	246 ± 23	197 ± 47	209 ± 43	7 ± 35	2.5 ± 12
$\Delta D_{tot}^{SP}$ (ms)	395 ± 73	214 ± 45	88 ± 19	66 ± 31	35 ± 11	4 ± 36	0.5 ± 5
$\tau_{D}^{SP}$ (ms)	173 ± 33	187 ± 42	167 ± 39	179 ± 55	183 ± 47	29 ± 43	12 ± 18
Average HR (bpm)	75.3 ± 0.7	62.6 ± 0.5	93.6 ± 0.7	110.9 ± 1	139.2 ± 1	0.2 ± 0.8	-
SP ratio (%)	54	60	85	77	92	-	-

*The normalized error ± standard deviation as well as the ratio of impulses passing through the SP are also presented*.

**Figure 6:**
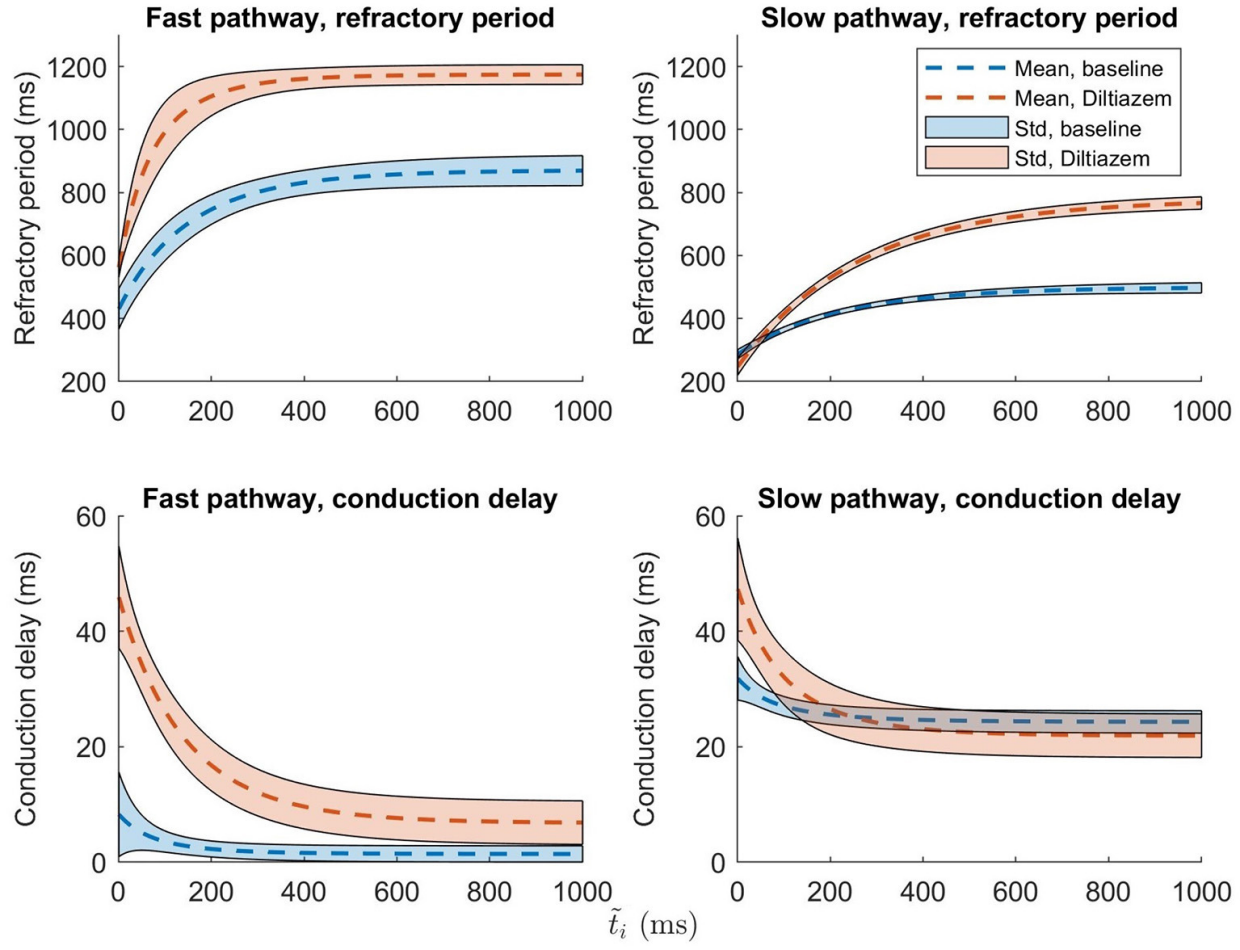
The mean ± one standard deviation, indicated by the shaded background, of the estimate refractory period and conduction delay from Equation (1) and (2), after 200 runs, are plotted for both baseline (blue) and Diltiazem (orange).

To set the robustness in perspective, the AV nodal properties were estimated 200 times for a single patient during baseline and under the influence of the non-dihydropyridine calcium channel blocker rate control drug Diltiazem. The results, shown in Figure 6, indicate that the uncertainty in the parameter estimation is sufficiently low in order to reveal the drug effect.

### 3.3. Model Comparison

**Figure 7:**
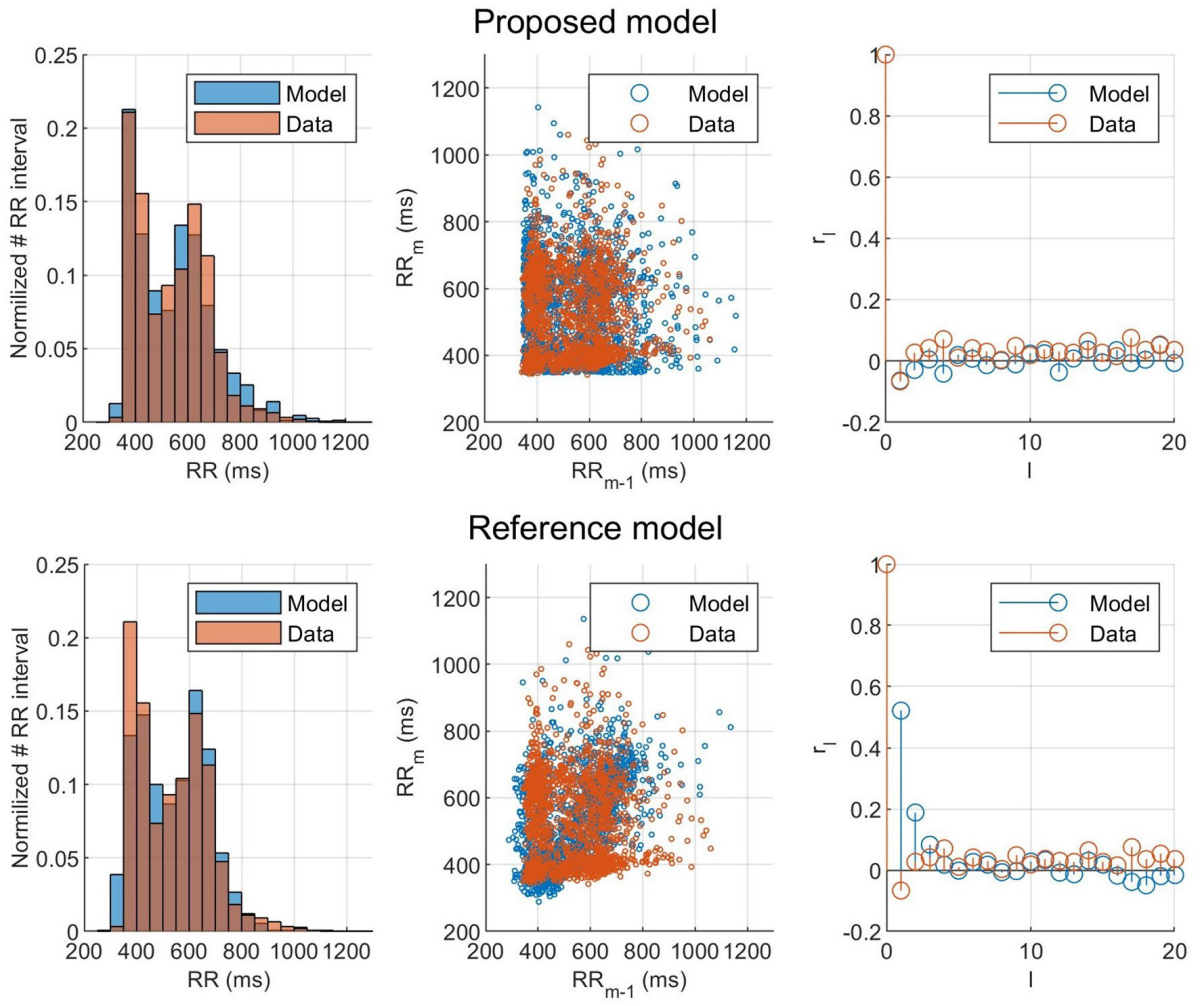
Histogram, Poincaré plot, and autocorrelation representation of the (orange) observed and (blue) modeled RR interval series for **(top)** the fitted proposed model and **(bottom)** the fitted reference model.

To evaluate the ability of the model and proposed workflow to represent AF data and to have a frame of reference, the proposed model is compared with the model presented in Wallman and Sandberg (2018); henceforth denoted the reference model. Both models were fitted to the RR interval series from one example patient, and the properties of the resulting simulated RR interval series are shown in the form of histograms, Poincaré plots, and autocorrelations, as seen in Figure 7. For both models, the optimizer was run until no change in error value for the fittest individual during ten generations occurred, to assure convergence. Both models used the optimizer described in section 2.5, but the reference model uses a fitness function based on the histogram (Wallman and Sandberg, 2018). It is clear from both the Poincaré plots and the autocorrelation plots that the proposed model can better replicate the dynamics of the RR interval series. The fit to the Poincaré plot can be quantified by the resulting ϵ, which for the proposed model was 1,360, compared to 6,740 for the reference model. Similarly, the value for the first lag autocorrelation was −0.07 for the proposed model and 0.52 for the reference, compared to the ground truth at −0.07.

## 4. Discussion

In this study, a mathematical model of the AV node, bundle of His, and Purkinje network has been presented together with a fitness function accounting for RR interval dynamics and genetic algorithm tailored to the model. The model and workflow have been evaluated with respect to robustness, accuracy, and ability to represent data, using both measured and simulated data.

Ten nodes in each pathway were used as a trade-off between detail and computation time. A small number of nodes can make the conduction delay larger than the refractory period, allowing impulses to bounce back and forth, whereas a large number of nodes leads to a higher computational demand. The inclusion of a last node in the model as functionally distinct from the SP and FP has previously been used in other models of the AV node (Inada et al., 2009). The incorporation of separate conduction properties for the connecting node introduced both new refractory period and conduction delay parameters. However, literature data suggests that inter-patient variability in conduction time over the bundle of His and the Purkinje network is around 10 ms (Deshmukh et al., 2000), indicating that the parameters representing the conduction delay could be reasonably approximated by a constant value. Furthermore, an initial study was conducted in which the refractory period of the HP node was represented by Equation (1), with three free parameters. This study showed that the parameter values representing the refractory period in the HP node found after optimization matched a constant value of *RR*_*min*_, independent of ${\tilde{t}}_{i}\left(n\right)$, well; indicating a good approximation (data not shown). For more details about the parameter values of the HP node during the optimizations, see Supplementary Section 2.

Reducing the number of free parameters reduces the parameter space in which the GA operates, and in turn decreases the running time as well as increases the robustness for the optimization. The parameters for the HP node were especially advantageous to fix or estimate directly from data. This was partly because the clinical data and analysis of the optimization made it possible, and partly because the most interesting information regarding the AV node is contained in the parameters governing SP and FP. Thus, setting the parameters corresponding to the bundle of His and Purkinje fibers to fixed values enhanced the ability of our method to estimate AV node properties.

The optimizer in this work utilized the fact that the model could be used with varying speed and precision by changing the output length, with higher speed and lower precision at the start and shifting it during the optimization. This change in output length also made it possible to run a broad search of the parameter space fast at the start of the optimization by restarting it several times; reducing the risk that a parameter set producing a good fit to the RR interval series was missed. This led to finding parameter sets matching the data faster, as shown in Figure 5. With a computing time of 20 min on a standard desktop computer in order to estimate the parameters, it possible to utilize the model without the use of any cloud computing or supercomputer, making it suitable for routine off-line analysis of Holter recordings.

The result of taking the RR interval series dynamics into account during the optimization can clearly be seen in Figure 7, where the proposed model and fitness function could represent the Poincaré plot with an ϵ five times as low as the reference model. This shows that matching the histograms well, as both models did, does not necessarily mean that the model represents the RR interval dynamics well. Using the Poincaré plot as basis for the fitness function, it was possible to account for the RR interval distribution and the one-step autocorrelation at the same time. It should be noted that the information from the histogram is still indirectly included in the Poincaré plot, which is likely the reason why the proposed fitness function also gave well matched histograms.

Since no ground truth of the estimated parameters is available for the clinical data, it is not possible to directly verify their correctness. However, it is still possible to verify that the parameter values lay within ranges reported in literature. The conduction delay for the HP node is fixed based on clinical data, thus it lies within reasonable ranges by default. The refractory period for the HP node was estimated using *RR*_*min*_, and for the five patients used in this study the range was [292, 655] ms. Comparing this to the bundle branch refractory period of [305, 520] ms, and the His-Purkinje system relative refractory period of [330, 460] ms, reported in Denes et al. (1974), it seems reasonable.

It is difficult to assess AV conduction delay during AF, due to problems in determining which atrial impulse activated the ventricles. However, the total minimum and maximum prolongation of conduction delay parameters of the AV node, $D_{min,tot}^{FP}$, $\Delta D_{tot}^{FP}$, $D_{min,tot}^{SP}$, and $\Delta D_{tot}^{SP}$, have previously been estimated by mathematical models utilizing the relationship between diastolic interval and delay in Equation (2). One such example is the model by Mangin et al. (2005), which uses invasive data, for which the ranges of *D*_*min,tot*_, Δ*D*_*tot*_, and τ_*D*_ were [80,300], [15,125], and [80,340], respectively. These ranges are of the same order of magnitude as the values obtained for *D*_*min,tot*_, Δ*D*_*tot*_, and τ_*D*_ in the present study, cf Table 2. It should be noted that the present model, contrary to the Mangin model, has two pathways where shorter delays are expected for the FP than for the SP.

The maximum refractory period, defined as the sum of *R*_*min*_ and Δ*R*, can be compared with electrophysiological measurements of the AV node effective refractory period. The values obtained in the present study were in the ranges [466, 973] and [257, 735] ms for the FP and SP, respectively. AV node effective refractory periods from patients with reentrant tachycardia have been reported in the ranges 361 ± 57 and 283 ± 48 ms for the FP and SP, respectively (Natale et al., 1994). As expected, the FP has larger values in both model and measurements.

The use of simulated data was necessary in order to have a ground truth to compare the estimated parameters with and in turn evaluate the methodology. From these five simulated data sets, it is clear that all of them primarily used the SP, cf. Table 2, although the SP ratio differed. This higher usage of the SP may be a contributing factor to that the parameters representing the SP were more accurately estimated than the parameters representing the FP. Moreover, the parameters $\tau_{R}^{SP}$, $\tau_{R}^{FP}$, $\tau_{D}^{SP}$, and $\tau_{D}^{FP}$ all have a larger error, which might imply that they have smaller overall effect on the model output. Further, histograms and Poincaré plots highlighting the transmission pathway for the RR intervals (cf. Supplementary Section 3) show that longer RR intervals tend to be transmitted via the FP, which is to be expected given its lower total conduction time. More interestingly, it is evident that different histogram peaks generated by the model are not created solely from one pathway, but stem from complex interaction between both the FP and SP. Moreover, it should also be noted that the difference in heart rate between the observed RR interval series and the RR series produced by the fitted model was less than one beat per minute.

It is evident from the example in Figure 6 that the uncertainty in conduction delay and refractory period introduced by the parameter estimation is generally lower than the effect of the drug, thus suggesting that it is possible to assess the effect of rate control drugs on the AV node from non-invasive data. For the example patient, the difference in conduction delay for the SP between baseline and Diltiazem is minimal for $\tilde{t_{i}}>200$ ms. However, one patient is not enough to know if this is a feature specific to this particular patient, a property of the investigated drug, or an artifact of the model formulation. The effect of rate control drugs on the AV node refractory period have previously been investigated (Sandberg et al., 2015), and with the proposed methodology a similar investigation can be done for AV node conduction delay.

### 4.1. Limitations and Future Work

The main limitation of the present study is the lack of comparison between the estimated parameter and the ground truth AV node characteristics, making the results more difficult to evaluate. Although simulated data was used as a substitute, it is not fully known how closely it matches reality. Another limitation is the assumption that both pathways are activated simultaneously, an assumption that may not be valid, since the electrical activity in the atria is highly disorganized. The variation in output originating from the stochastic input sequence can also be seen as a limitation to the proposed model, since the output for a single set of parameters can vary depending on the realization of the input sequence. However, without electrical measurements in the atria, it is not possible to model the exact behavior of the AV node.

Moreover, due to the computational time of estimating the parameters for each simulated RR interval series 200 times, only a subset of RATAF was used. However, the five patients were selected to ensure a representative subset based on their RR interval series characteristics. It should be noted that the focus of the present study is to evaluate the robustness in parameter estimation rather than analysis of the RATAF data set. Using the model to analyze the entire RATAF data set, including all patients, drugs, and time segments for outcome prediction forms a natural next step in this line of inquiry, and efforts toward this goal are ongoing at the time of writing.

Example results, cf. Figure 6, suggest that the estimates of refractory period and conduction delay are sufficiently robust to detect changes in response to treatment with rate control drugs. However, this needs to be verified in a larger study population. By using the model to simulate the treatment effect of different drugs in a patient-specific setting, it might be possible to predict the outcome of the drug treatment and thus assist in treatment selection. Furthermore, it could also be useful in drug development, by aiding in understanding what AV node properties are affected by a novel compound, and in what way.

## 5. Conclusion

We have described and motivated a network model of the AV node, bundle of His, and Purkinje network. The model is demonstrated to be able to represent RR interval series extracted from ECG data well, both in the forms of histograms, Poincaré plots, and autocorrelation. This was made possible using the presented problem specific fitness function and optimization algorithm, taking advantage of the model's ability to increase running speed at the cost of precision. The robustness in parameter estimation enabled fitting of delay specific parameters from the AV node solely based on the ECG. It also made it possible to detect changes to the model parameters originating from the use of a rate control drug.

In summary, the combination of model and parameter estimation workflow presented here constitutes a significant improvement on previous AV node modeling efforts, suggesting the possibility to use ECG measurements to analyze drug effect on the AV node on a patient specific level.

## Data Availability Statement

The simulated data supporting the conclusions for this article will be available from the authors upon request. The measured data are owned by Vestre Viken Hospital Trust, and requests for access can be made to Sara R. Ulimoen. The code for the model together with an user example can be found at https://github.com/FraunhoferChalmersCentre/AV-node-model.

## Ethics Statement

The studies involving human participants were reviewed and approved by the Norwegian Medicines Agency. The patients/participants provided their written informed consent to participate in this study.

## Author Contributions

MK, FS, and MW contributed to conception and design of the study. SU gathered and organized all raw data. FS computed and organized all RR interval series and λ. MK designed the changes to the model as well as the genetic algorithm with advice, suggestions, and supervision from FS and MW, and wrote the manuscript. FS and MW supervised the project and revised the manuscript during all the writing process. All authors contributed to manuscript revision, read, and approved the submitted version.

## Funding

This work was supported by the Swedish Foundation for Strategic Research (Grant FID18-0023), the Swedish Research Council (Grant VR2019-04272), and the Crafoord Foundation (Grant 20200605).

## Conflict of Interest

The authors declare that the research was conducted in the absence of any commercial or financial relationships that could be construed as a potential conflict of interest.

## Publisher's Note

All claims expressed in this article are solely those of the authors and do not necessarily represent those of their affiliated organizations, or those of the publisher, the editors and the reviewers. Any product that may be evaluated in this article, or claim that may be made by its manufacturer, is not guaranteed or endorsed by the publisher.
