# Supplementary material for: Non-invasive Characterization of Human AV-Nodal Conduction Delay and Refractory Period During Atrial Fibrillation
Source: Front Physiol. 2021 Oct 28;12:728955. doi: 10.3389/fphys.2021.728955 (PMC8584495; doi:10.3389/fphys.2021.728955)
Supplement: Supplementary file 1 [file Data_Sheet_1.PDF]

Supplementary Material

1 GENETIC ALGORITHM

The selection for the genetic algorithm (GA) was performed using tournament selection, with two participants selected randomly from the current population. From these two participants the fittest, i.e., the one with the lowest error value (ϵ), was selected with a probability of P_{Tour} (Table S1). Otherwise the less fit one was selected. This was completed two times to select two parents that were used for the crossover. However, crossover was only performed with a probability of P_{Cross} (Table S1). Otherwise the parents survived into the next stage. Before crossover was performed, the Euclidean distance between the parents was determined and if it was lower than $Incest_{Max}$ (Table S1) the two parents were seen as too similar and two new parents were selected with the same tournament selection as described previously.

A two-point crossover was then performed, where the two points were randomly selected from the range $[1, N_{Para}]$, where N_{Para} is the number of parameters. The two parents were then split up and the different selected subsets of parameters were switched with each other. The two new individuals were then sent to the next stage; mutation.

The new individuals were selected for mutation with a probability of $P_{Mut,Ind}$ (Table S1). Individuals that were not selected were transferred to the next generation unchanged. If an individual was selected for mutation, each parameter was selected for mutation with a probability of $P_{Mut,Para}$ (Table S1). When a parameter was selected for mutation, the new parameter value was selected with creep mutation, using a normal distribution with the mean value at the current parameter value. The standard deviation for this normal distribution was the selected parameter's value range, i.e. the difference between the maximum and minimum value for the parameter during the initialization, multiplied by $Gain_{Std}$ (Table S1). Thus, mutations were more likely to cause small changes in parameter values. This process of selection, crossover, and mutation was iterated until there were as many new individuals as in the previous generation (N_{Pop}) minus the number of elite individuals, i.e. the fittest 2.5% from the last generation. The elite were always transferred to the next generation unchanged.

The value of $P_{Mut,Para}$ at the start of the optimization was $\frac{2}{N_{Para}}$, so that on average two parameters were selected for mutation. However, this varied depending on the diversity (D) of the population, defined as

$$D = \frac{1}{N_{Pop}(N_{Pop}-1)} \sum_{i=1, i\neq j}^{N_{Pop}-1} \sum_{j=1}^{N_{Pop}} d(i,j) \tag{S1}$$

where

$$d(i,j) = \frac{1}{N_{Para}} \sum_{k=1}^{N_{Para}} \frac{|I_{i,k} - I_{j,k}|}{R} \tag{S2}$$

where $I_{i,k}$ is the i:th individual in the populations k:th parameter, and R is the range which again is the difference from the maximum and minimum value that parameter could have during the initialization. If the diversity was higher than D_{Max} (Table S1), the mutation rate $P_{Mut,Para}$ was decreased by division with D_{Change} (Table S1). If $P_{Mut,Para}$ was lower than D_{Min} (Table S1), however, $P_{Mut,Para}$ was increased by multiplication with D_{Change}. This ensures that the diversity of the population is high enough to decrease the risk of premature convergence.

All parameter values used in the GA can be found in Table S1, and the choice of these values will now be explained. The values of P_{Tour} and P_{Cross} were set at standard values for a GA Wahde (2008). $Incest_{Max}$ was set based on how much the parameters could change while still resulting in approximately the same model output, defined as an average parameter change of 2.5%. The $P_{Mut,Ind}$ was set to a high number, so that the majority of the individuals were mutated. As mentioned before, the value of $P_{Mut,Para}$ at the start of the optimization is $\frac{2}{N_{Para}}$, so that on average two parameters were selected for mutation. Two out of twelve parameters on average are normally seen as very high Wahde (2008), and was selected to avoid premature convergence. D_{Min} was set by checking the diversity during several runs of simulated patients and seeing when the algorithm started to converge prematurely, and thus needed a higher mutation rate. D_{Max} was set by checking when the high mutation rate started to make the GA too slow in finding fit individuals. The D_{Change} value was set so that the mutation rate changed very quickly if the diversity was not between D_{Min} and D_{Max}, since the algorithm does not run for many generations, meaning that a small D_{Change} might not have time to affect the diversity. All values for D_{Min}, D_{Max}, and D_{Change} were set closely to values previously reported in literature Wahde (2008). The $Gain_{Std}$ was set so that too large mutations were very unlikely, since very large mutations were found to almost always result in high values of ϵ.

Table S1. All the parameters that can be changed in the genetic algorithm and their values.

Parameters	Value
P_{Tour}	0.75
P_{Cross}	0.75
$Incest_{Max}$	0.025
$P_{Mut,Ind}$	0.8
$P_{Mut,Para}$	Varying with start at $\frac{2}{N_{Para}}$
$Gain_{Std}$	0.05
D_{Min}	0.2
D_{Max}	0.3
D_{Change}	0.2

2 REFRACTORY PERIOD ESTIMATION

Before deciding on using RR_{min} as a fixed value for the refractory period for the HP node, an initial study was conducted where the HP node was represented with the same equation as the other nodes, Equation 1 in the main article, using the three parameters R_{min}^{HP}, ΔR^{HP}, τ_R^{HP}. However, during parameter optimization of the model, it was observed that the value of ΔR^{HP} tended to be very low, leading to a low impact on the model outcome. This implies that $R_i^{HP}(n)$ is independent of $\tilde{t}_i(n)$, staying constant at R_{min}^{HP}. This in turn makes the time constant τ_R^{HP} superfluous. Thus, ΔR^{HP} and τ_R^{HP} were both set equal to zero. Moreover, the value of R_{min}^{HP} tended to be very close to the minimum RR-sequence in the data. Therefore, it was deemed sufficient to estimate the value of R_{min}^{HP} directly from the RR interval series instead of including it in the optimization. The results of this initial study can be seen in Table S2.

Table S2. The mean and standard deviation of R_{min}^{HP} and ΔR^{HP} with the minimum RR interval series for comparison.

Patient #	R_{min}^{HP} (ms)	RR_{min} (ms)	ΔR^{HP} (ms)
1	505 ± 19	499	24 ± 25
2	650 ± 25	655.3	99 ± 32
3	416 ± 11	414.2	43 ± 27
4	351 ± 7	348.4	16 ± 15
5	233 ± 28	292	105 ± 51

3 VISUAL REPRESENTATION OF THE SIMULATED DATA

The histogram and Poincaré plots for the simulated RR interval series resulting from the parameter sets in Table 1, with the conducting pathway marked, are shown in Figure S1 and Figure S2, respectively. The effect of changing only the arrival rate of atrial impulses, λ, can also be seen in Figure S1.

REFERENCES

Wahde, M. (2008). *Biologically inspired optimization methods: an introduction* (WIT press)

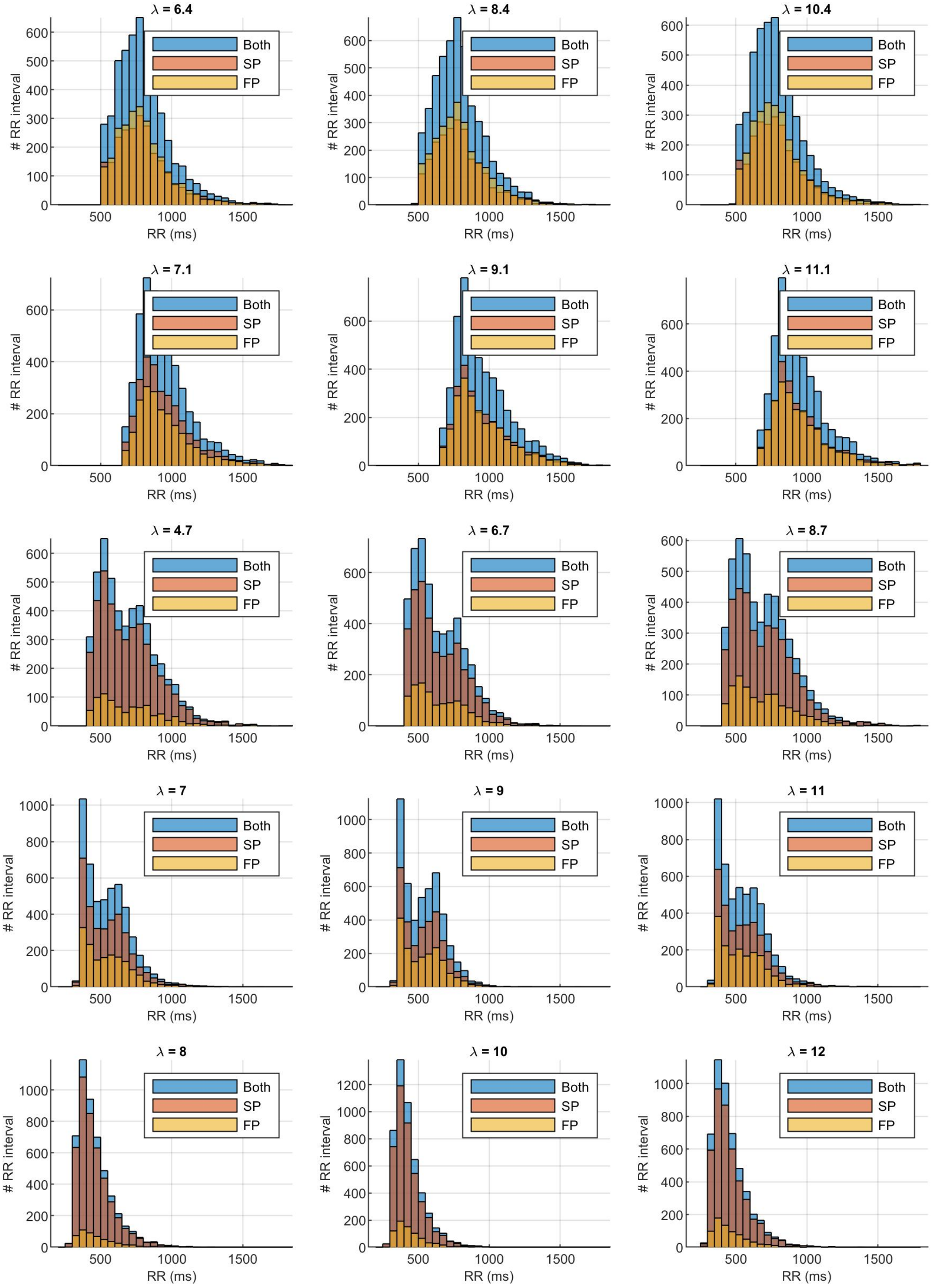

Figure S1. RR interval histograms based on all conducted impulses (blue), impulses conducted through the SP (red), and impulses conducted through the FP (yellow) resulting from simulations with the five parameter sets defined in Table 1 (middle column), and with decreased (left column) and increased (right column) values of lambda, respectively. The parameter sets are ordered by number, with 'Patient 1' in the top panel and 'Patient 5' in the bottom panel.

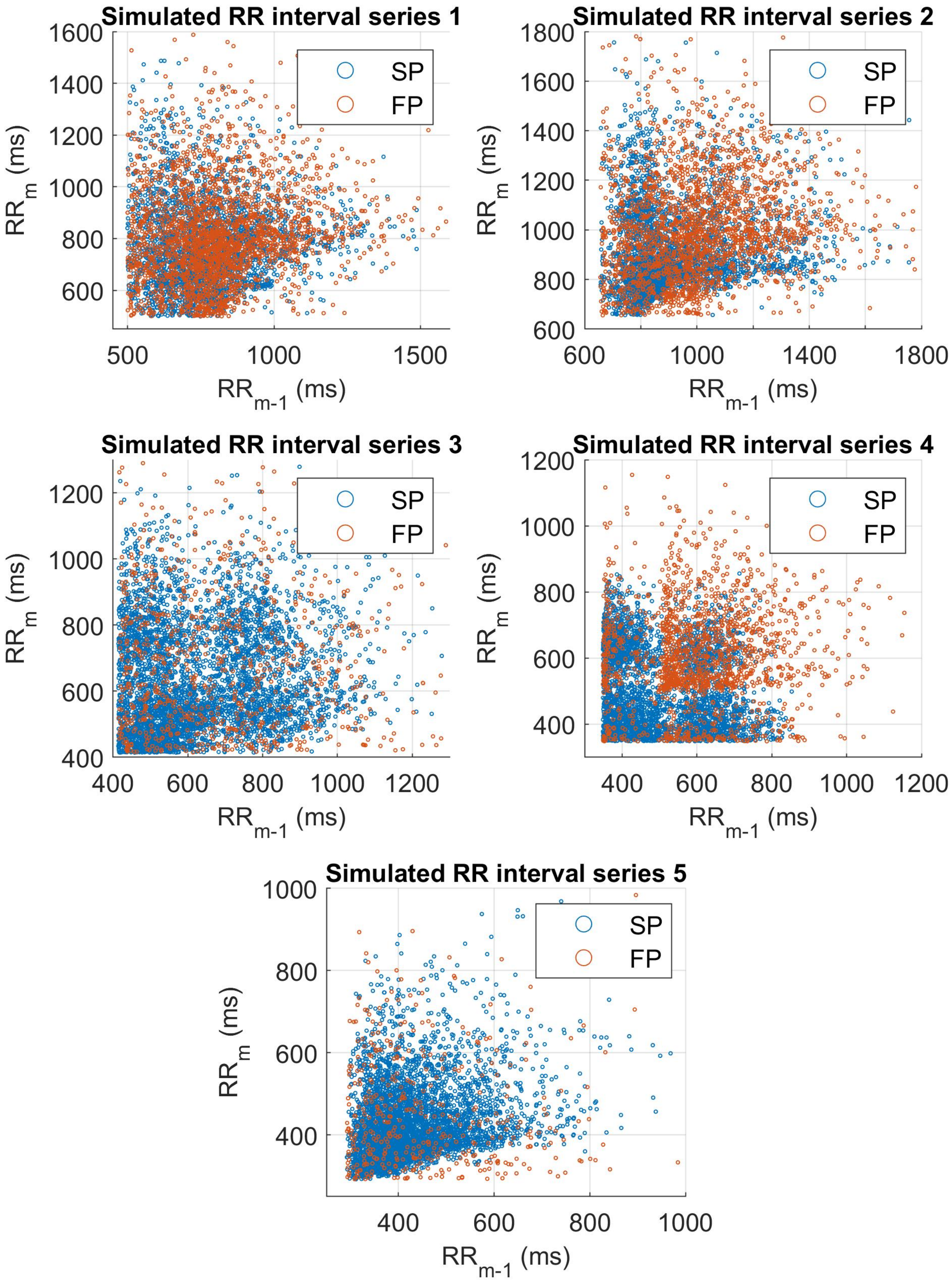

Figure S2. Poincaré plots based on impulses conducted through the FP (orange), and impulses conducted through the SP (blue) resulting from simulations with the five parameter sets defined in Table 1.
